# The Eye Hospitals of Vienna

**Published:** 1873-01

**Authors:** A. W. Calhoun

**Affiliations:** Vienna, Austria


### FOREIGN CORRESPONDENCE.

### THE EYE HOSPITALS OF VIENNA.

BY DR. A. W. CALHOUN.

In a certain sense, the establishment of institutions for the sick and unfortunate more clearly marks the prosperous progress of those communities in which such homes are founded, than any other step toward the general good. There is no necessity for an argument to illustrate that the poorer classes become victims to disease at least as often, if not oftener, than those more profusely provided for. And also is argument unnecessary to make known the fact, that as rapidly as cities advance in wealth and population, equally as rapidly do they fill with that class whose ills are a constant demand upon the authorities for a display of humanity and generosity. But few cities of any importance refuse or ignore these demands. On the contrary, they meet them in such a liberal and substantial manner—viz., by founding hospitals—that, dismissing all other considerations, it becomes an internal strength and support—

giving aid to the suffering, removing a burden from the city, inviting within its borders especially that class with moderate means, who come knowing that, should misfortune and sickness overtake them, a home is always awaiting them, in which the truest and most scientific efforts are expended for their sound and rapid recovery, and so, in short, contributing to the general prosperity of all.

Viewing it simply in a philanthropic light, it is with the utmost pleasure and admiration that one beholds the interest and maintainance given to such institutions in all European countries, not only by separate communities, but also by the government itself; each apparently vieing with the other in the relief of their fellow-sufferers. Equally as much as this zeal for hospitals among Europeans is to be admired, just so much is the indifference or neglect of our own people in this particular to be deplored. But they are now moving in this matter—prompted, perhaps, by their own interest, as well as for humanity's sake, and it is hoped that something desirable will be attained before the work is finished.

Though general hospitals have been known for years, yet it is within comparatively recent years that hospitals, or portions of hospitals, have been to any extent devoted exclusively to the treatment of special diseases. This practice is even on the increase, and as medical men are continually developing new specialties, whose importance increases with their development, so new wards and new hospitals for the treatment of special affections are being constantly established. So it has been for a long time with eye diseases, and also more recently with those diseases affecting the throat, the ear, the urinary organs, etc.

As Vienna is at present the center at which all students of ophthalmology congregate, and where the most celebrated teachers in this speciality are daily to be heard, it will probably not be inappropriate to give a few facts in reference to the eye hospitals under the charge of these luminaries in the profession, together with their management, etc.

No doubt but that all physicians, and especially those who are acquainted with the foreign medical literature, have time and again heard something of the immense extent of the departments in the different hospitals of Vienna, devoted alone to the eye, and the incomparable advantages to be derived from an attendance upon these special clinics. It is scarcely possible that any reports could easily exaggerate their importance and benefits, but to properly appreciate them requires a visit and inspection from those interested in such institutions and studies.

The three leading spirits in this branch of medicine are Profs. Arlt, Jaeger and Stellway: the latter well known in America as the author of a most valuable and interestingly written work on the eye, the English translation of which has for many years been a "Text Book," as it were, amongst American students of opthalmology; the second, justly known as the finest and most successful ophthalmoscopic teacher of the present day; and the first, Prof. Arlt, now regarded as the Graefe of Europe.

The portion of the general hospital occupied by Prof. Arlt, consists of four large and conveniently arranged apartments, aside from others used for examining patients, lecture and clinical room, etc. In these wards are ninety beds, exclusively for the use of patients who have been operated upon, or for those whose diseases are of so grave a nature as to demand their reception into the hospital. Unfortunately these wards are not sufficient, for individuals whose cases need immediate attention are daily turned away with the answer that not a vacant bed is to be had.

During the year just gone, including two months' holiday, when but little is being done, about three hundred cataract operations were made, and even now, the very season that such operations are less seldom performed, from the fact that cataract patients are erroneously impressed with the belief that any operative interference during the winter is apt to result disastrously, it is still being daily made to greater or less extent. This refers to cataract alone, for during the same period near eight hundred general operations were made, including only those whose proper treatment required the patients to remain for a time in bed. Such operations as strabismus and others similar, are not classed in this reckoning, as these were afterward treated in the daily clinic.

An excellent idea is practiced in reference to operating in the wards. Where any of the severer operations are to be made, the patient is placed upon the bed in which he is to remain, and the operation made as required. This certainly simplifies the many details requisite under such circumstances, and dispenses entirely with the trouble, inconvenience, and also the danger of transporting a patient to and from an operating room to a place of quiet and comfort. Strict attention to such apparently small points contributes no little to the enormous per centage of recoveries after operations of this kind in these wards. It is no wonder that success follows so promptly, when an operation so carefully considers all the minutiæ, and has first class assistants and well trained nurses to put them into execution.

Unlimited opportunities are given the student in watching every step of each case as it progresses from day to day, favorably or otherwise, and the different modes of treatment employed under differcnt circumstances or emergencies, for full freedom is allowed in making the daily visits, morning and afternoon with the assistants, who more than willingly explain any dark points in connection with the different cases as they pass under treatment.

It is now a generally recognized fact, learned from experience, that the most impressive manner of teaching medicine in any or all of its branches, is to bring the student in direct contact with the sick, and thus with the disease to be contended against, whereby he may readily distingush and overcome his foe before he reaches a shape and size beyond control. This is fully appreciated here by all engaged in teaching, and particularly in this special eye department, where the Professor, in addition to and between the regular visits, makes a tour of the wards, with and for the special benefit of the large class under his charge.

The old idea of keeping the wards of an eye hospital in an absolutely darkened condition, has long since been exploded, it having been found not only of the greatest inconvenience, but altogether unnecessary. Of course the bright light is carefully excluded, but the rooms are never so dark but that the convalescents can freely move about and attend to such pastime work as their condition will permit.

Most surprising however, is to visit the ambulatorium or general clinic, in which are daily treated the lighter diseases, whose violence is not so great but that the patients can attend to their various occupations, or at any rate return regularly to the hospital for treatment, etc., and in the meantime manage their own cases at home. Such numbers and the dispatch with which they are treated, at the same time making each group of cases a sort of special study before the class, and such a satisfactory manner of teaching is found only in Vienna. An idea may be formed of the importance of these clinics, when I mention that the cases deemed worthy of being registered have during this year numbered over five thousand, not mentioning those slight troubles, embracing many hundred, which with the first visit were dismissed as cured. As may be inferred from what has been before said, this is exclusive of all operations, such as extraction or discision of cataract, iridectomy, enucleation of bulbus, operations upon the lids, and many others of equal importance. With the new cases of each day, and those returning regularly for treatment, there is passed in review before the class a daily average of between fifty and sixty patients, often reaching nearer a hundred, containing every variety of disease from simple conjunctivitis to the well-to-be-dreaded glaucoma, and embracing every period of life, from the new-born babe to extreme old age, typical specimens of each being from time to time discussed, and the most rational treatment put into use. A regular attendance upon these clinics and witnessing the multitude of afflictions to which the eye is heir, continually demonstrates what a broad and inexhaustible field of study this organ is, containing manual work for the most skillful and practical hands, and dark and obscure points which have engaged the time and thought of some of the most scientific minds of the age, and who still have many interesting and necessary problems yet to solve. Could some of these *anti-specialists* take even a cursory look into many of these departments for special affections, they could readily convince themselves of the utter impossibility of all of the branches of medicine being *crammed* into any one brain.

As an evidence of the neglect that many separate organs have hitherto suffered at the hands of even the most learned in the profession, who have adopted general and frowned at special studies, we have but to call to mind the most wonderfully rapid and surprisingly important progress these organs have received, even almost from the first moment of their adoption as specialties. No better example of this can be offered than the eye, which—from once being a subject misunderstood, and hence mistreated, and deemed unworthy of more than a partial thought from the busy professional man—has, in rather recent years, become so developed that it now consumes the whole time and attention of many of the brightest lights in the profession. The thankful service that the labors of these specialists have and are still rendering to mankind, convinces me of the fact that the science might be still more surely and rapidly advanced, and many of the professional hindrances and obstructions corrected and dissipated, were others of our colleagues induced to adopt, for special and exclusive study, many of those organs of the economy, whose perfect and clear comprehension as to function and disease has by no means as yet been reached.

Next in size and consequence come the wards of Professor Jaeger, consisting of eighty-two beds, constantly filled with intensely instructive cases of severe disease, or such resulting from the varied and numerous operations, and offering the most interesting and endless study to the oculist. What was said of visiting the wards of Professor Arlt, holds also good here—the same plan being practiced in both departments, and the same privileges being allowed to those taking an interest in such work. For what is called "outside treatment," more than four thousand cases have visited his clinic during the present year, again embracing every conceivable species of eye trouble.

Can the amount of knowledge to be acquired by four hours' daily attendance upon such clinics (for each continues two hours and often much longer)—and still others, if desired—be over-estimated, more particularly since what we see and hear is actually practical—that which we are continually in need of in our every day private practice. The different modes of operating to reach the same object are constantly performed, more especially to practically demonstrate them and their different effects to the student, and thus we are afforded actual results by which to form opinions as to the most appropriate and suitable plans to be followed in the removal of different diseases. During the past twelve months, near six hundred operations have been made, which include those for cataract, iridectomy, strabismus, etc.—in short, all those practiced upon the eye. Of these, near two hundred have been for cataract. I spoke of the formerly prevailing idea of having the wards totally darkened as having been discarded, as is the case at Professor Arlt's; but I notice that Professor Jaeger, although perhaps the originator of the change, has not entirely forgotten the old rule or habit, for amongst his many apartments one is thoroughly darkened, in which to place those to whom he thinks such care and caution requisite. The other rooms are only so arranged as to avoid the effects of a strong and clear light, as in the other hospital. In main, the same customs and regulations are practised in the two departments, and the same advantages offered by one are also found in the other.

Altogether separate from these, are the wards of Professor Stellway—being a portion of the military hospital—the Professor himself being, if I am not misinformed, a military surgeon. Although established for the use and benefit of those in military service, they are now open for the public, as those of Arlt and Jaeger. The reputation of such a man is enough to insure full clinics on every occasion. But the people, although accustomed from youth to contact with the military, and also to military service at a later period of their lives, rather reluctantly place themselves, when suffering from disease, in the hands of military surgeons for treatment, and only the well-known and highly appreciated abilities of Professor Stellway as an occulist induce patients to a crowded attendance upon his clinics, notwithstanding the prejudices above mentioned. Still they have their effect, and hence the somewhat smaller number of eye patients to be seen daily in his clinics than in those before mentioned. However, that the students of eye diseases understand the benefits of his clinical teachings is evinced by his daily filled lecture room. I have not been able to procure the exact number of clinical patients, or of the operations for the last year; but it is sufficient to say that the first amounts to several thousand, and the second to many hundred.

Aside from all these, there is another institution, known as "*the polyclinic*," which differs from the others inasmuch as no operations are here performed, and only those cases treated, not only not demanding operative assistance, but who are in condition to return for daily treatment. Whenever their severity extends beyond this point, or the use of the knife is necessary to save the eye, the cases are referred to one or the other hospitals for reception and treatment. Even in this "polyclinic," the patients are also several thousand annually, and afford the best of all opportunities for close inspection of the different cases, since fewer visitors are present, it having no connection with the University, as do the other departments.

These are the public clinics alone, and when summed together make an imposing array—viz., about ten thousand clinical cases annually, *exclusive* of the clinics of Professor Stellway and the "polyclinic," these increasing the number several thousand more, but whose exact account I failed to receive—between one and two thousand of the most important operations performed upon the eye, including near six hundred for the removal of cataract. Certainly an inviting field is here open to those engaged in such studies—a field whose extent and fertility finds no comparison in any European city.

London has also considerable reputation as an ophthalmic school, but after a few months' trial, most of the students abandon that city and come to this, not simply on account of the greater number of cases to be seen, but also on account of the infinitely superior facilities possessed by the Vienna schools in reference to teaching the many subjects associated with a complete knowledge of the eye. This special manner of teaching is also peculiar only to Vienna.

Private courses in ophthalmoscopy are given daily by certain of the assistants, and also one by Prof. Jaeger himself, in all of which students are thoroughly taught the proper use of the ophthalmoscope, which comparatively, in the last few years has become of such indispensable aid in diagnosing ophthalmic diseases. In these courses we have set before us every imaginable variety of these affections seated in the interior of the eye, and are taught how to investigate and to recognise them as to position, nature, etc. Material for such investigation is inexhaustible, and here we grasp ideas and facts that are in future to prove invaluable to many a sufferer, threatened with such a blow as loss of vision.

Courses on refraction and accommodation, one of the most difficult, and at the same time one of the most essential studies in ophthalmology, and one equally as enticing as that referring to any other organ of the body, are delivered, and so to speak, greedily attended. This is no easy work, requiring many and repeated strokes to be brought within easy comprehension, but the zeal with which the hearers push forward, indicate their determination to become masters of this subject so absolutely necessary to a full knowledge of the physiological action of the interior apparatus of the eye, which, for so long a time remained a mystery to us. To such men as Donder and Helmholz, are due a world of thanks that they have brought from darkness to light, and clearly demonstrated many of these intricate problems which for years and years had baffled the genius of a host of others.

Teaching the various operations and their different modes of performance is by no means neglected. Private classes under the assistants and one under Prof. Jaeger, offer all that one can desire in this particular. Performing general operations upon the cadover as a good practice for surgeons, cannot be too highly estimated, but more especially is this true in respect to the delicate operations upon the eye, which require the utmost precision and delicacy in their different steps to insure success. Operating material being in such sufficiency, these courses constitute amongst others, a prominent feature in Vienna eye schools.

More recently have been commenced courses for examining microscopically the pathological changes of the eye, a need of which has so long been felt, but few oculists in Germany giving as yet special attention to this study, essential to an understanding of the multiform changes undergone in the eye during disease.

Noteworthy is the great number of Americans at present in Vienna, studying the eye as a specialty. It not only indicates a need of such information amongst our American surgeons, but exhibits the growing tendency in the profession to adopt specialties, induced both by a desire to better advance by this means the general science, as well as to make their own private practice more pleasant and lucrative. At the present rate, but a few years will suffice to make specialists of all kinds as numerous in America as they are over the whole of Europe. But to this no objections should be urged, since the field is immense and work exists enough for all. In this way is the practice of physicians narrowed into defined limits, their labors lightened, the science of medicine rapidly advanced, and human suffering infinitely more readily subdued.

VIENNA, AUSTRIA, November 9, 1872.

